# Effects of High vs. Low Glycemic Index of Post-Exercise Meals on Sleep and Exercise Performance: A Randomized, Double-Blind, Counterbalanced Polysomnographic Study

**DOI:** 10.3390/nu10111795

**Published:** 2018-11-18

**Authors:** Angelos Vlahoyiannis, George Aphamis, Eleni Andreou, George Samoutis, Giorgos K. Sakkas, Christoforos D. Giannaki

**Affiliations:** 1 Department of Life and Health Sciences, University of Nicosia, 46 Makedonitisas Avenue, Nicosia CY 1700, Cyprus; angelvlahogiannis@windowslive.com (A.V.); aphamis.g@unic.ac.cy (G.A.); andreou.el@unic.ac.cy (E.A.); 2 University of Nicosia Medical School, 93 Agiou Nikolaou street, Nicosia CY 2408, Cyprus; samoutis.g@unic.ac.cy; 3 Faculty of Sport and Health Sciences, University of St Mark & St John, Plymouth PL68BH, UK; gsakkas@marjon.ac.uk

**Keywords:** post-exercise nutrition, sleep, polysomnography, visual reaction, sprint interval training

## Abstract

The aim of the current study was to investigate the effect of the glycemic index of post-exercise meals on sleep quality and quantity, and assess whether those changes could affect the next day’s exercise performance. Following a baseline/familiarization phase, 10 recreationally trained male volunteers (23.2 ± 1.8 years) underwent two double-blinded, randomized, counterbalanced crossover trials. In both trials, participants performed sprint interval training (SIT) in the evening. Post-exercise, participants consumed a meal with a high (HGI) or low (LGI) glycemic index. Sleep parameters were assessed by a full night polysomnography (PSG). The following morning, exercise performance was evaluated by the countermovement jump (CMJ) test, a visual reaction time (VRT) test and a 5-km cycling time trial (TT). Total sleep time (TST) and sleep efficiency were greater in the HGI trial compared to the LGI trial (*p* < 0.05), while sleep onset latency was shortened by four-fold (*p* < 0.05) and VRT decreased by 8.9% (*p* < 0.05) in the HGI trial compared to the LGI trial. The performance in both 5-km TT and CMJ did not differ between trials. A moderate to strong correlation was found between the difference in TST and the VRT between the two trials (*p* < 0.05). In conclusion, this is the first study to show that a high glycemic index meal, following a single spring interval training session, can improve both sleep duration and sleep efficiency, while reducing in parallel sleep onset latency. Those improvements in sleep did not affect jumping ability and aerobic endurance performance. In contrast, the visual reaction time performance increased proportionally to sleep improvements.

## 1. Introduction

There is a growing interest among the scientific community towards the potential effects of sleep on both athletes’ health and exercise performance. Interestingly, recent data revealed that 30–50% of adult athletes are subject to poor sleep [1,2,3]. Although it is difficult to discriminate between psychological burden and actual physical overreaching, it was demonstrated that sleep quality may be compromised by high-intensity training sessions [4]. High-intensity exercise training has become very popular not only among competitive athletes but also in the general population and in even patients with chronic diseases [5].

In addition, sleep is a significant part of post-exercise recovery process, since an inadequate amount of sleep hinders physical and mental recovery after strenuous exercise [6,7]. During nocturnal sleep, metabolic and endocrine functions (i.e. release of growth hormone), coordinate to promote adequate body relaxation and tissue restoration [8]. Thus, sleep after exercise training or before an athletic event plays a pivotal role in achieving sufficient recovery levels in order to prepare the athlete for the upcoming event, while potential sleep disturbances may be detrimental for athlete performance [7,9]. Consequently, several studies have been conducted in order to evaluate the effectiveness of strategies such as cold water immersion [10] and light exposure [11] as a means of improving athletes’ sleep. Nevertheless, the efficacy and practicality of such interventions remain questionable. 

Another strategy that has been under investigation to improve sleep is nutritional interventions. Occasionally, assorted nutritional interventions have been tested to improve sleep parameters in a sedentary population. These include the combination of tart cherries and apple juice as a mean to increase the dietary consumption of melatonin [12] or exogenous tryptophan (Trp) supplementation to promote in vivo synthesis of melatonin, which plays a major role in the regulation of sleep–wake cycles [13]. Another promising intervention to enhance sleep is carbohydrate consumption. Since 1981, Porter and Horne have found that a pre-bed meal with high carbohydrate content leads to more restful sleep [14]. Additionally, carbohydrate quality (i.e., glycemic index) was found to reduce sleep onset latency via its effect on increasing blood tryptophan levels [15]. However, trials of such interventions have been conducted in a sedentary population, over a short-term period during which the participants had to refrain from strenuous physical activity. Therefore, there is lack of available data on the effects of post-exercise carbohydrate interventions on sleep-related parameters during periods of intensive exercise training.

Additionally, most of the nutritional interventions implemented over the years to optimize athletic performance adjust carbohydrate intake via modifications on their quality (i.e., glycemic index) and quantity of consumption [16,17]. Carbohydrates are related to several body functions, such as brain function, and contribute as a fuel during the majority of athletic events [18]. As an ergogenic aid, carbohydrate manipulations are usually used to determine their direct effect on performance and muscle glycogen replenishment. Although the contribution of carbohydrates to exercise performance is well-established, the effects of post-exercise carbohydrates with respect to sleep-related parameters and the subsequent effects on exercise performance the following morning have not yet been investigated.

Based on literature review, integrative approaches combining the monitoring of post-exercise nutrition, sleep and their subsequent effect on exercise performance are understudied. Thus, the current study aims were two-fold. Firstly, the aim was to evaluate the effect of the glycemic index of post-exercise meals on sleep-related parameters in a real-world case scenario of an evening high-intensity exercise session. The second aim was to further investigate whether this nutrition intervention and its subsequent effect on sleep-related parameters could, in turn, be translated into benefits for exercise performance-related components such as power, reaction time, and aerobic endurance performance (cycling) the following morning, as is the case in multi-day sport events.

## 2. Materials and Methods

### 2.1. Participants

Ten recreational-trained male volunteers (23.2 ± 1.8 years) were recruited for this study. Inclusion criteria were the following: male subjects from 18 to 30 years old, who had been exercising at least thrice a week for the past two years with workouts including high-intensity training. Exclusion criterion included any kind of history of major chronic disease or the taking of any medication. Before preliminary exercise testing, all participants were examined by a medical doctor and obtained clearance for participation in the study. All participants were informed for the purposes of the study and provided a written consent form. The study was approved by the Cyprus National Bioethics Committee (Project ID: EEBK/EΠ/2018/10) and all of the procedures were conducted according to the manual of the Declaration of Helsinki in 1964 and its later amendments.

### 2.2. Study Design

**Figure 1:**
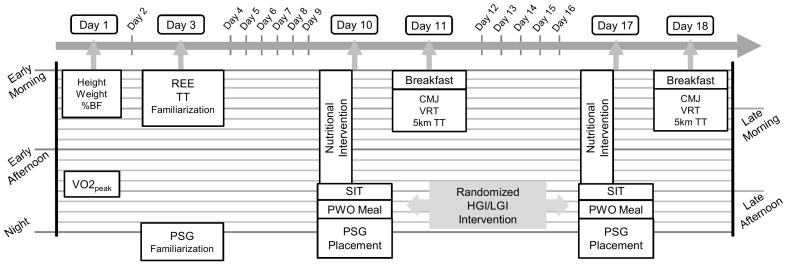
Schematic overview of study design. TT: time trial; REE: resting energy expenditure; PSG: polysomnography; CMJ: countermovement jump; VRT: visual reaction time, TT: time trial; PWO: post-workout; HGI: high glycemic index, LGI: low glycemic index; VO_2peak_: peak oxygen uptake; SIT: sprint interval training.

Initially, participants underwent a baseline/familiarization phase to obtain basic somatometric and anthropometric data, resting energy expenditure (REE), and peak oxygen uptake (VO_2peak_). In a double-blind, randomized, and counter balanced crossover design, the post exercise meal’s glycemic index (GI) was tested for its effect on sleep parameters and following morning’s exercise performance. A schematic overview of the study design is depicted in Figure 1. Randomization of crossover was determined by a computer-generated sequence of random numbers. The nutritional intervention designed in that way so that the participants could not distinguish the two conditions. The scientist who analyzed the polysomnographic data was not aware of the study’s trial sequence. Similarly, the researchers were blinded to intervention for the testing of exercise performance.

During each intervention, participants were asked to abstain from any intense physical activity and followed a personalized nutritional plan. On trial day, in the evening, the participants arrived at the laboratory at around 18:00 h and exercised on stationary bike, following a sprint interval training (SIT) protocol (described further below) under constant supervision. Following exercise, the participants were provided with a standardized meal with either high glycemic index (HGI) or low glycemic index (LGI). Two hours later, participants underwent a sleep study (polysomnography) in their home. The following morning, participants were given a standardized breakfast. Three hours later, the participants visited the laboratory to assess exercise performance. A seven day wash out period separated the two trials. Each trial took place on exactly the same day of the week and at the same time of day.

### 2.3. Preliminary Testing

The first visit to the laboratory was used for preliminary testing. Weight and standing height were measured to the nearest 0.1 kg and 0.5 cm, respectively, with a Tanita WB-3000 digital beam scale (Tanita Corp, Tokyo, Japan). Body fat percentage (% BF) was estimated via Harpeden skinfold caliper using Jackson’s and Pollock’s 7-site method [19]. For this technique, skinfold measurement of specific body areas of the right side of subjects was measured twice from the same researcher. If the two values deviated more than 5%, the measurement was repeated one more time and the average value of the measurements was recorded. Body fat percentage was calculated from body density according to Siri’s equation.

The same day in the afternoon, VO_2peak_ was assessed on a cycle ergometer (Monark LC6, Monark Exercise AB, Vansbro, Sweden). The height of the saddle and handlebar were adjusted individually for each subject and remained the same for all training and performance-testing procedures on the stationary cycle ergometer. The initial workload of the incremental VO_2peak_ test was set at 50 W and kept increasing by 25 W every minute until the participants reached volitional exhaustion or could not maintain a cadence of 60 revolutions per minute (rpm), and respiratory exchange ratio (RER) was above 1.1. Oxygen uptake was measuredbreath-by-breath by a metabolic cart (Quark CPET, Cosmed, Rome, Italy). Heart rate was continuously monitored with a Polar heart rate monitor (Polar® H7, Polar Electro Oy®, Kempele, Finland). VO_2peak_ was defined as the mean VO_2_ over the last 10s of the exercise test. The maximum power output (W_max_) achieved during the incremental cycling test was later used to determine exercise intensity during the SIT protocol.

The second visit to the laboratory took place 48–72 h later, after an overnight fast to measure their REE with indirect calorimetry (Quark CPET, Cosmed, Rome, Italy) in a dark room. Subjects lay quietly on a bed for 20 minutes prior to a 20-min gas collection to determine REE. The first and the last five minutes of gas collection were discarded from the analysis [20]. After REE determination, the participants were familiarized with the 5-km time trial (TT) and were then given nutritional advice, which is described extensively in the corresponding section further below. On the following evening of preliminary testing the participants underwent a familiarization trial of the polysomnographic study procedure.

### 2.4. Nutritional Intervention and Assessment of Dietary Intake

After preliminary testing, all participants received dietary instructions for the following 14 days so that a balanced and controlled nutritional plan was adopted and no individual was under a special diet throughout the study. Caffeinate beverages, nutritional supplements and alcohol were not permitted for 48h prior to the sleep study and the following morning. Post-exercise/pre-bed meal and breakfast were prepared and given by a registered nutritionist/dietitian.

In particular, subjects followed the same isoenergetic nutritional plan during the two trials, based on their measured REE, physical activity level, and calories burned during exercise. A mean daily energy consumption was 2523.2 kcal ± 182.7, providing 1.5 g ± 0.2·kg^1^ of protein and 5.5 g ± 0.8 kg^1^ of carbohydrates.

Three hours prior to the evening SIT exercise protocol, participants consumed their pre-workout meal, which consisted of 200 g of low-fat yogurt (2%) and one banana (approximately 100 g). Immediately following exercise, participants consumed one of the test meals high in carbohydrates, prepared by the researcher and differentiated for glycemic index (GI), as described by Afaghi et al. [15]. Both test meals contained a combination of steamed rice (either parboiled (low GI:52) or jasmine (high GI:109)) [21] and vegetables, supplying approximately 2 g kg^1^ carbohydrates. Glycemic load (GL) was calculated as ((GI/100) × g available carbohydrate) [15]. The GL was approximately 81 and 170 for the low-and high-GI meals, respectively. For preparing rice, water was boiled and then 200 g or raw rice were added, this was allowed to boil for another 15 min. Parboiled rice gives a weight ratio of 1:2.25 and jasmine rice gives a ratio of 1:3.05 raw to boiled weight. Participants were supervised in order to ensure full consumption of the test meal.

The following morning, 2 h before exercise performance testing, all participants drank 600 mL of water and consumed a standardized meal consisting of 370 mL of semi-skimmed milk, 45 g of breakfast corn flakes, and two bananas (approximately 200 g), a meal high in carbohydrates and low in fat (483 kcal; 102 g carbohydrate; 17.9 g protein, and 3.3 g of fat).

### 2.5. Sprint Interval Training Regime

The SIT exercise session took place in the laboratory under the supervision of an exercise physiologist. On both experimental visits to the laboratory, first the participants warmed-up by cycling for 10min at an intensity of 35% W_max_. Then, they performed six repetitions of 20 s “all out” bouts at 140% W_max_ separated by 140 s of active recovery by cycling at 20% W_max_. Heart rate was monitored during the SIT session, at the beginning, ending and five seconds after the end of each sprint using a Polar heart rate monitor. Exercise sessions ended with 10-minute post exercise active recovery cycling at 50% of W_max_.

### 2.6. Polysomnography

Polysomnographic study (PSG) took place on three occasions; familiarization and two main trials at the participants’ home, using a portable PSG device (Somnoscreen, Somnomedics GmbH, Randersacker, Germany). Usual bedtime was assessed from the familiarization trial. Time to bed was consistent for each trial and sleep duration was ad libitum. During the main trials the participants were asked not to take any nap because of its effect on nocturnal sleep patterns [22].

The PSG study was performed in random order according to the guidelines of the American Academy of Sleep Medicine (AASM) [23]. The set-up of the examination included the application of the following: electroencephalogram (F3, F4, C3, C4, P3, P4, O1, O2, ground (at AFz) and a reference electrode at position FCz) right and left electrooculogram; submental and tibial electromyogram; body position; electrocardiogram; thoracic and abdominal efforts (piezoelectric transducers); heart rate and oxygen saturation.

Sleep stages and arousals determined using the criteria of the AASM [23]. For each sleep period, the following variables were calculated: total sleep time (TST, i.e., the amount of sleep time whilst in bed); sleep stages N1, N2, N3, and rapid-eye movement (REM) as a percentage of TST (the N1 and N2 stages are often denoted as “light sleep” and N3 as “deep sleep”, and the N1, N2, and N3 stages are all denoted as non-REM (NREM) sleep);% sleep efficiency (SE, i.e., TST/time in bed × 100); sleep onset latency (SOL, i.e., the time elapsed from laying down until N1 stage sleep onset); REM onset latency (i.e., the time elapsed from laying down until REM stage sleep onset); deep sleep onset latency (i.e., the time elapsed from laying down until N3 stage sleep onset); total wake time (i.e., the total amount of wakefulness from N1 stage sleep onset until the final awakening); and the arousal index (i.e., the number of arousals per hour,) during the REM stage, NREM stages, and in total.

The PSG examination performed to the participant’s home in order to maintain as possible their sleep routine and environment. The researcher arrived at the participant’s home and following set up of the device, the participants went to bed at their habitual time, as indicated from the first familiarization sleep study. The PSG examination was terminated the following morning. In the case scenario of failure to obtain sleep study-related data, the participants were asked to repeat the study.

### 2.7. Exercise Performance Testing

The following morning the participants visited the laboratory againin order to perform the tests related to exercise performance, including a countermovement jump (CMJ), a visual reaction time (VRT) test and the time to complete a 5-km time trial (TT).

Both the CMJ and the VRT tests were performed using an optoelectonic measurement system (Optojump Next, Version 1.3.20.0, Microgate, Bolzano, Italy). After five minutes of cycling at 35% of W_max_ for warm-up, the participants performed three CMJs (1-min rest between jumps) and the highest one was recorded.All jumps were measured in cm to the nearest 0.1 cm. Afterwards, a visual reaction test (VRT) was performed using a computer screen and the Optojump measurement system. Subjects stayed at semi-squat position with their hands on their hips. Immediately after receiving a visual stimulus at the computer’s screen, the participants had to perform a squat jump with hands on hips as quickly as possible and as high as possible. This test consisted of three jumps initiated by the visual stimuli with 1 min rest between trials. The reaction time after each stimulus was recorded at the nearest 0.001 s.

Finally, a 5-km TT was performed on a stationary bike (Monark LC6, Monark Exercise AB, Vansbro, Sweden). Volunteers self-selected the cycling intensity in order to cover 5km as fast as possible. Heart rate was recorded during the test by a heart rate monitor. On the bike’s screen, every information about heart rate, watt or rpm were hidden, except for a bar that was fulling proportionally to their process to end the test. Two buttons to increase or decrease workload (Watts) were used by the subjects to adjust their cycling speed, according to their perceived exertion. Once the test was completed, the subjects underwent a 10-minute cool-down by cycling at 50% of W_max_.

### 2.8. Statistical Analysis

Data are reported as mean ± standard deviation. Statistical analysis was performed with IBM®SPSS® statistics for Windows, version 22.0 (IBM Corp, Armonk, NY, USA). A Shapiro–Wilk test was conducted to identify variables’ distribution. In order to compare means between trials we used paired samples *t*-test or Wilcoxon test for parametric and non-parametric variables, respectively. Cohen’s d_z_ effect size was calculated and the 95% confidence interval (lower, upper) computed to illustrate the magnitude of change. Potential correlations explored with Pearson’s r or Spearman’s correlation coefficient. The alpha level for all statistical analyses was set at *p* < 0.05, with two-tailed tests.

## 3. Results

### 3.1. Preliminary Data

Participants’ age was 23.2 ± 1.8 years and the body fat percentage was 12.7± 5.1%. Mean VO_2peak_ was 44 ± 6.8 mL·min^−1^·kg^1^. The maximal heart rate during VO_2peak_ testing reached 177 ± 15 beats per minute (bpm) and the corresponding load ranged from 220 W to 320 W, with a mean value of 260 W.

### 3.2. SIT-related Data

**Figure 2:**
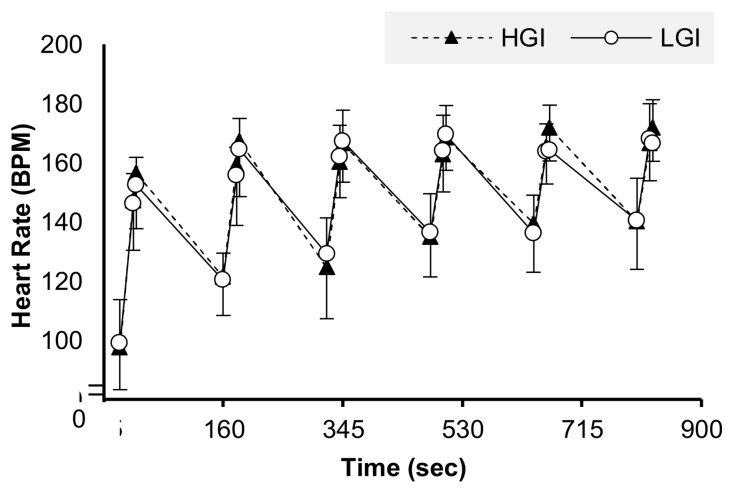
Heart rate during the evening SIT exercise in the HGI and LGI trial.

During SIT training, individuals reached 97% and 96% of the HR_peak_ (obtained during the VO_2peak_ test) in the HGI and LGI trials, respectively. The HR before and after the SIT protocol was similar in both of the main trials (Figure 2).

### 3.3. Polysomnography-Related Data

Mean values for all sleep parameters are shown in Table 1. Total sleep time (TST) was significantly longer after the post-exercise HGI meal compared to the LGI trial (*t*(9) = 2.9, *p* = 0.019, 95% CI (13.18,111.76) d_z_ = 0.9). In the HGI trial, sleep efficiency (SE) was higher than in LGI trial (*t*(9) = 2.3 *p* = 0.049, 95% CI (0.04,16.50) d_z_ = 0.72). Sleep onset latency (SOL) as initially found to be decreased by three-folds in the HGI trial compared to the LGI trial. However, further analysis showed that the SOL results were affected by an outlier (3.4 SD above mean). After removal of the outlier only for this analysis, the SOL in the HGI trial found to be decreased statistically significantly by four-fold compared to the LGI trial (*t* (8) = 2.7, *p* = 0.026, 95% CI (−44.65, −3.72) d_z_ = 0.90). No significant differences were found for the various sleep stages. Total wake time was higher in the LGI compared to HGI trial (*t* (9) = 2.5, *p* = 0.034, 95% CI (3.17,63.46) d_z_ = 0.79). Arousal index (the number of arousals per hour) during REM, NREM, and total sleep time did not differ between conditions. 

### 3.4. Exercise Performance Data

**Figure 3:**
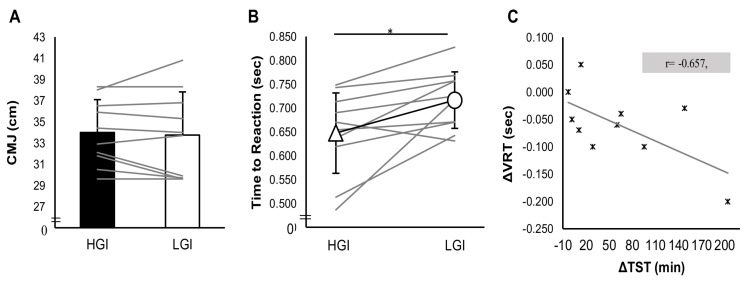
Exercise performance in (**A**) countermovement jump test; (**B**) visual reaction test, between trials, and (**C**) the correlation of the difference between HGI and LGI total sleep time with the difference between the HGI and LGI visual reaction test. * Denotes statistical significant differences at the 0.05 level (2-tailed).

Countermovement jump performance did not differ significantly between trials (*p* > 0.05) (Figure 3A). Performance on VRT found to be improved on HGI trial comparing to LGI trial with a large effect size. In particular, reaction time was significantly less in HGI trial comparing to LGI trial (*t*(9) = 2.9 *p* = 0.018, 95% CI (−0.107, −0.013) d_z_ = 0.91,) (Figure 3B). Performance in the 5-km time trial did not differ between trials (*p* > 0.05). However, a statistically significant difference was found between heart rate at the start and end of the time trial. Starting and ending heart rate were higher in the HGI trial compared with the LGI trial (*t*(8) = 2.9, *p* = 0.019, 95% CI (2.06, 17.28) d_z_ = 0.98; *t*(8) = 3.3, *p* = 0.01, 95% CI (2.21, 12.23) d_z_ = 1.10) (Table 2).

### 3.5. Correlations Between Sleep-Related Parameters and Exercise Performance-Related Parameters

Deep sleep onset latency was positively related with minimum visual reaction time (*r* = 0.489, *p* = 0.033). No statistically significant correlations were found between the rest of the examined variables (Table 3). The potential relationship of sleep extension, which reported in HGI trial, with VRT was further investigated. For this reason, the difference between the TST of the HGI trial and TST of the LGI trial (ΔTST) were computed. Accordingly, the difference between the VRT of the HGI trial and the VRT of the LGI trial (ΔVRT) was estimated. These variables showed statistically significant collinearity, as shown in Figure 3C.

### 3.6. Statistical Power

Post hoc analysis reveals that with *n* = 10, the statistical power for the primary outcomes such as sleep time and sleep efficiency was 80% (*a* = 0.05, effect size = 1.00, *t* = 2.25) and 82% (*a* = 0.05, effect size = 1.03, *t* = 2.26), respectively.

## 4. Discussion

To our knowledge, this is the first study to investigate the effect of a post-high intensity exercise nutritional intervention on both sleep quality and quantity and next day exercise performance. The findings of the current study reveal that a HGI post-exercise meal matched for caloric and carbohydrate intake extends sleep duration by 17%, improves sleep efficiency by 8.1%, and reduces SOL approximately by 4-fold compared to a LGI post-exercise meal, as measured by the gold standard method of full-night polysomnography. Additionally, a moderate to strong correlation was found between the amount of sleep extension that was achieved in the HGI trial and the corresponding improvements in the visual reaction test the following day. In contrast, improvements in sleep were not translated into significant enhancements of either jumping ability or aerobic endurance performance.

Carbohydrate manipulations are the most common interventions in the sports nutrition field and vary among different sports [16]. The majority of studies focused on the effects of carbohydrate quantity during post-exercise recovery indicate that low carbohydrate consumption slightly decreases REM sleep and increases parallel deep sleep [14,24]. Nevertheless, there are a limited number of relevant studies investigating the effect of glycemic index on sleep [6], and no study had previously assessed the effect of the composition of post-exercise/pre-bed meal on sleep parameters and sleep architecture.

The findings of the current study did not show any significant effect of GI on sleep quality, confirming previous data [15]. Afaghi and colleagues in 2007 showed that consuming a similar meal four hours prior bedtime results in decreased SOL [15]. In line with the above findings, in the present study it was found that when a similar meal was used as a post-exercise meal led to increases in sleep duration, improvements in sleep efficiency, and most importantly, a reduction in SOL in recreationally active males.

Since an increasingly percentage of athletes experience sleep issues [1,2,3] and there is an indication of an acute sleep deterioration after high-intensity training [4], a HGI carbohydrate intervention after evening high-intensity exercise should be considered of great importance for levering out those abnormalities. This intervention may act as a safeguard against health risks and daytime sleepiness which are both induced by sleep deprivation [9], with a further benefit for athletes’ performance. While most studies focus on the potentially negative effects of sleep deprivation on exercise performance, the results of this study encourage the investigation of the beneficial effects of sleep optimization on exercise performance (i.e. visual reaction time).

The HGI intervention of post-exercise meal resulted in significant improvements in sleep by prolongation of sleep, increments on efficiency and beneficial reductions in SOL and total wake time. To some extent, these results are equivalent to the sleep-related effects of exogenous melatonin administration. In non-athletic populations, melatonin is proven to act beneficial on sleep initiation and maintenance [25]. A meta-analysis conducted in 2013 [25] showed that melatonin ingestion by individuals with sleep disorders extended total sleep time, decreased sleep onset latency, and improved overall sleep quality. Expanding these results to a sample of young athletes, melatonin administration after late-night exercise prolonged sleep and improved sleep efficiency, whilst reducing SOL and total awake time after sleep initiation [26]. 

Identification of the underlying physiological mechanism leading to reduced SOL following the HGI meal is beyond the scope of the present study. However, certain assumptions can be made. It can be hypothesized that HGI resulted in an increase in the postprandial insulin levels. As insulin affects metabolism of protein, carbohydrates and lipids, the ratio of Trp to large neutral amino acids (LNAA) in plasma will be raised [27]. Tryptophan competes the other LNAAs for transport across the blood–brain barrier; hence, an increased ratio of Trp:LNAA is related to increases in brain Trp [28]. More tryptophan entering the brain will lead to greater production of 5-HTP and serotonin, and finally secretion of melatonin, resulting in sleepiness [29,30]. Notably, as a single bout of SIT training increases insulin sensitivity [31], it may implied that exercise influences the extension of these physiological responses to HGI meals and the beneficial effect on sleep-related parameters.

In the current study, the restorative effects of sleep were showed directly through the improvements in VRT performance. In particular, time to reaction was reduced by 8.9% in the trial with the HGI nutritional intervention as compared to the LGI trial. Numerous studies show that cognitive performance and especially time to reaction are affected predominantly by sleep length [32,33,34]. When sleep is compromised by reducing its duration, vigilance (as a state of alertness) is decreased significantly [33]. Additionally, sleep extension interventions from two to six weeks have resulted in improved concentration and reduced reaction time from 4.3% to 11.7% in both rugby and basketball players, respectively [34,35]. It is important to note that the current study showed that even a short-time intervention that optimized sleep was capable of inducing remarkable benefits in VRT. Important to highlight is the fact that the amount of sleep expansion was linearly related to the subsequent improvements in VRT.

Another performance-related parameter that was evaluated in the present study was lower body power as expressed by the performance in the CMJ test. In line with the results of the present study, Fullagar and colleagues [36] showed that improvements in sleep quantity via sleep hygiene interventions had no effect on CMJ performance. Acute sleep restriction appears not to impair CMJ height in athletes [36,37], while vertical jump may be compromised during periods of extreme sleep deprivation [38]. Countermovement jump execution may differ among studies and it is observed that when CMJ is executed with hands on hips, there are no deviations in jumping height performance [36,37], while with a vertical jump test with an arm swing, like a “reach and touch”, there is a moderate impairment after sleep restriction [38]. From this point of view, sleep appears not to interfere with lower body power but might play a key role in neuromuscular junction and coordination. On the other hand, there is no evidence that acute sleep extension improves CMJ height.

Aside from cognitive performance and lower body power generation, endurance exercise requires the integration of multiple physiological systems to modulate performance. In the present study, time to complete the 5-km TT did not differ between nutritional interventions. Acute carbohydrate interventions are considered critical for substrate storage, utilization, and adequacy during endurance exercise [16]. Still, the effects of the post-exercise meal’s GI on prolonged exercise are controversial [39]. The results in the present study support the work of previous research conducted by Ormsbee and colleagues [40], who found that a pre-night feeding with chocolate milk did not improve performance in a 10-km time trial in competitive female runners and triathletes after regular nocturnal sleep. When total sleep deprivation occurs, the performance of aerobic exercise that lasts approximately 60 min is impaired [41]. Accordingly, partial sleep restriction after an exhaustive evening training session decreases performance in a 3-km TT [42]. Thus, the present study indicates that there is no bidirectional relationship between sleep and short-distance aerobic endurance performance, since sleep optimization was not translated into improvements in the 5-km TT.

Overall, the scope of this study was to obtain an applicable intervention for athletes and individuals who exercise with high intensities with regard to the critical issue of nutrition and sleep to optimize recovery and exercise performance. The findings of the present study highlight the need to assess the quality of post-exercise carbohydrate consumption in order to maximize both recovery and exercise performance. It is notable that the current intervention supported the integral recovery process by preserving substrate restoration and optimizing sleep. The practical application of this intervention could be crucial for athletes that experience sleep disturbances during preparation, prior to, after, or during multi-day competitions [43,44] and for athletes who practice sports that require quick reaction times, such as soccer, basketball, etc.

The present study has several strengths, since personalized nutritional and exercise plans were provided to the participants and performance and sleep parameters were measured with gold standard methodology. However, additional strength performance tests, such as the isokinetic dynamometer, could have been used for further exploration of the effect of sleep on lower body power. Likewise, the evaluation of aerobic endurance performance with a test of different type or duration would be interesting. Due to financial constraints, we were unable to provide all daily meals to participants, and no hematological or histological modulations were explored.

To summarize, this is the first study to show that a post-high intensity exercise nutritional intervention improved both sleep and next day time to reaction but did not affect short-distance aerobic endurance performance and CMJ height. An HGI meal extended sleep time, leading to improvements in sleep efficiency and decreases in wake time and SOL. Furthermore, when a HGI meal was consumed post-workout, the next day’s VRT was significantly faster. Its practical application reflected in a real-world case scenario of evening training and following morning exercise performance testing and may possibly extend beyond recreational trained individuals to athletes of various levels that face sleep issues, or even to clinical populations.

## Figures and Tables

**Table 1:** Sleep-related parameters.

	HGI Trial	LGI Trial	*p* value
TST (min)	426.0 ± 71.7	363.6 ± 49.0	0.019 *
N1 (%)	14.2 ± 8.8	15.2 ± 10.4	0.059
N2 (%)	34.5 ± 13.1	33.4 ± 9.8	0.741
N3 (%)	20.8 ± 10.8	22.7 ± 13.3	0.495
REM (%)	28.6 ± 10.9	28.8 ± 12.2	0.959
SE (%)	89.0 ± 4.3	80.9 ± 10.6	0.049 *
SOL (min)	5.7 ± 1.9	24.6 ± 8.1	0.026 *
REM onset latency (min)	55.9 ± 39.4	84.8 ± 51.8	0.286
Deep sleep onset latency (min)	57.2 ± 66.8	62.6 ± 44.6	0.132
Total wake time (min)	47.4 ± 21.7	80.3 ± 43.6	0.034 *
Arousal index (number/h)			
REM	22.6 ± 8.6	22.1 ± 12.1	0.871
NREM	16.4 ± 7	18.4 ± 10.1	0.371
Total	18.5 ± 6.8	19.9 ± 10	0.239

All data are presented as mean ± SD. TST: total sleep time; REM: rapid-eye movement sleep; NREM: non rapid-eye movement; SE: sleep efficiency; SOL: sleep onset latency, * Denotes statistical significant differences at the 0.05 level (2-tailed).

**Table 2:** Performance parameters in the 5-km TT test.

	HGI Trial	LGI Trial	*p* value
5-km TT (sec)	461.6 ± 45.3	475.4 ± 53.3	0.441
W_avg_	143.9 ± 35.7	145.0 ± 35.7	0.877
W_max_	212.5 ± 84.3	221.5 ± 61.3	0.621
RPM_ave_	98.6 ± 10.3	95.6 ± 11.5	0.444
RPM_max_	129.3 ± 20.5	129.0 ± 24.2	0.963
HR_start_	99.7 ± 20.7	91.7 ± 17.2	0.019 *
HR_ave_	152.2 ± 14.6	148.4 ± 12.8	0.189
HR_max_	173.9 ± 10.4	169.4 ± 10.1	0.077
HR_end_	173.3 ± 10.4	166.0 ± 10.0	0.010 *

All data are presented as mean ± SD. TT: time trial, W: Watt, RPM: revolutions per minute, HR: heart rate. * Denotes statistical significant differences at the 0.05 level (2-tailed).

**Table 3:** Correlation coefficients between sleep-related and exercise performance-related parameters.

Exercise Performance-Related Parameters	CMJ (cm)	VRT_min_ (sec)	VRT_max_ (sec)	VRT_average_ (sec)	5 km TT (min)
Sleep-Related Parameters					
TST (min)	−0.277	−0.051	−0.093	−0.171	−0.055
N1 (%)	0.276	−0.002	−0.155	−0.086	−0.033
N2 (%)	−0.137	−0.2	−0.29	−0.278	−0.114
N3 (%)	0.091	−0.03	0.101	0.085	0.149
REM (%)	−0.08	0.197	0.303	0.247	0.073
SE (%)	0.103	−0.222	−0.089	−0.108	0.023
SOL (min)	−0.118	0.417	0.367	0.329	0.186
REM onset latency(min)	−0.182	0.208	0.24	0.289	−0.002
Deep sleep onset latency (min)	−0.237	0.489 *	0.321	0.361	0.012
Total wake time (min)	−0.068	0.247	0.159	0.206	−0.121
Arousal index (no/h)					
REM	0.325	−0.005	−0.148	−0.128	−0.004
NREM	0.148	0.123	0.063	0.07	0.105
Total	0.229	0.125	0.004	0.02	0.067

TST: total sleep time; REM: rapid-eye movement sleep; NREM: non rapid-eye movement; SE: sleep efficiency; SOL: sleep onset latency; CMJ: countermovement jump; VRT: visual reaction time, TT: time trial. * Denotes statistically significant correlations at the 0.05 level (2-tailed).
